# Magnifying endoscopic findings of early-stage poorly differentiated colorectal adenocarcinoma: a case report

**DOI:** 10.1186/s12876-022-02209-w

**Published:** 2022-03-28

**Authors:** Haiyan Li, Yao Liu, Jianhong Zhu

**Affiliations:** 1 Department of Gastroentorology, The Second Affiliated Hospital of Soochow University, Suzhou, Jiangsu Province China; 2 Department of Pathology, The Second Affiliated Hospital of Soochow University, Suzhou, Jiangsu Province China

**Keywords:** Colorectal poorly differentiated adenocarcinoma, Magnifying endoscopy, Chromoendoscopy, De-novo colorectal cancer, Case report

## Abstract

**Background:**

Colorectal poorly differentiated adenocarcinoma is rarely founded, especially in early-stage. Endoscopic features of early poorly differentiated colorectal cancer in magnifying endoscopy and chromoendoscopy haven’t been clarified.

**Case presentation:**

A 49-year-old man was referred to our hospital for endoscopic treatment of a lateral spread tumor located in the rectum. We performed pre-resection endoscopic examination for the patient. In magnifying endoscopy with crystal violet staining, the lesion showed irregular microvessels and turned out to be poorly stained with predominantly non-structural pit pattern and a few roundish pits scattered on the surface. The histology revealed a poorly differentiated adenocarcinoma of the rectum invading the deep submucosal layer with negative lymphovascular invasion.

**Conclusions:**

In this case report, we presented a case of poorly differentiated colorectal adenocarcinoma detected at an early stage, showing interesting endoscopic findings in magnifying endoscopy with crystal violet staining.

## Background

Colorectal poorly differentiated adenocarcinoma is rarely founded, especially in early-stage. Most cases are detected at an advanced stage. In contrast to differentiated adenocarcinoma, poorly differentiated adenocarcinoma often correlates with more aggressive biological behavior and is defined to be out-of-indication for endoscopic resection even in early-stage. Thus, the endoscopic diagnosis of poorly differentiated adenocarcinoma is important. Several endoscopic diagnostic classifications, including Kudo’s pit pattern classification, have been proposed and proved to distinguish colorectal cancer from non-neoplastic lesion or adenomas, as well as predict the depth of invasion in colorectal cancer. These diagnostic methods mostly focus on adenomas or differentiated adenocarcinomas. Perhaps due to its rarity, endoscopic features of early poorly differentiated colorectal cancer in magnifying endoscopy and chromoendoscopy haven’t been clarified. We here present a case of poorly differentiated colorectal cancer in the early-stage, showing specific findings in magnifying endoscopy with crystal violet staining.

## Case presentation

**Fig. 1:**
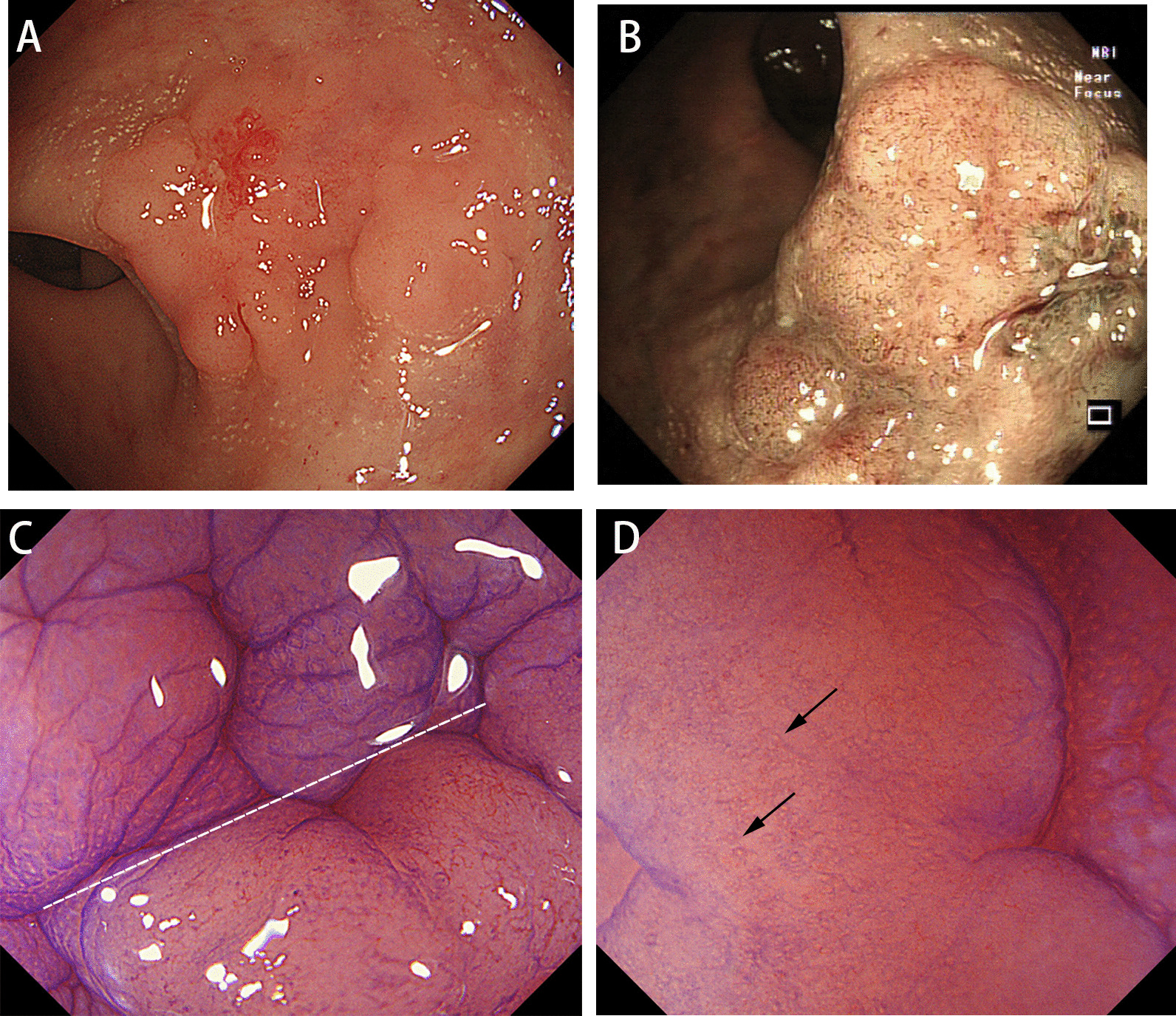
**A** White light endoscopy revealed a lateral spread tumor in the rectum. **B** In near focus NBI mode, the proximal part of the lesion presented with dense irregular microvessels. **C** In magnifying endoscopy combined with 0.05% crystal violet staining, the proximal part of the lesion showed poorly stained with predominantly non-structural pit pattern, while the background mucosa showed regular Type-I pit patterns according to the Kudo’s classification. The demarcation line was clearly visible (white dotted line). **D** In magnifying endoscopy combined with 0.05% crystal violet staining, the distal part showed poorly stained with predominantly non-structural pit pattern and a few roundish pits (black arrow) scattered over the surface

**Fig. 2:**
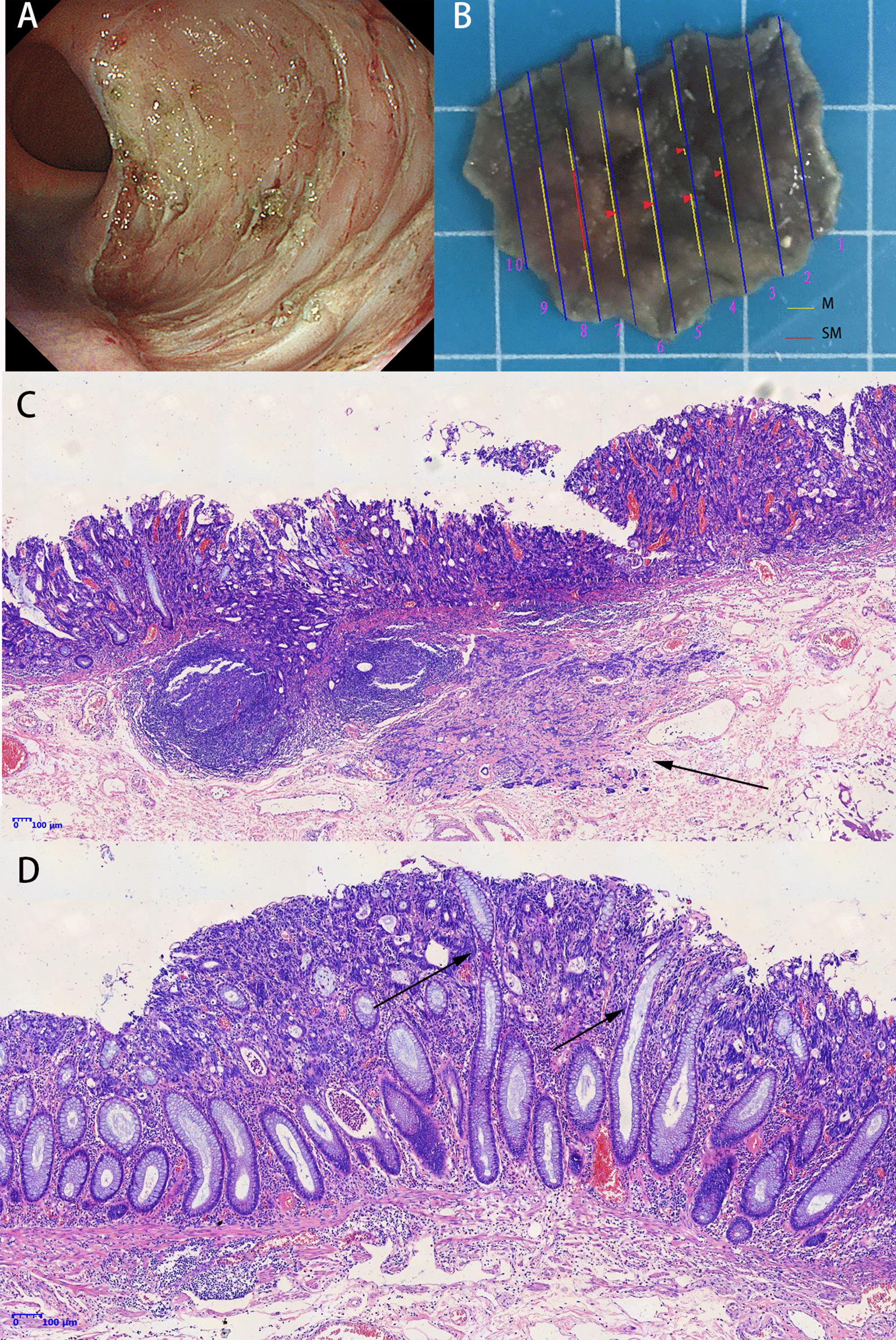
**A** The lesion was en bloc resected. **B** The specimen was sectioned at 2 mm intervals. **C** Histology showed poorly differentiated colorectal cancer with partial submucosal infiltration (black arrow). Stain: hematoxylin and eosin. **D** In some sections, histology showed a few normal glandular ducts (black arrow) surrounded by tumors cells, which is corresponding to the endoscopic feature, i.e., small roundish pits scattered over the surface

**Fig. 3:**
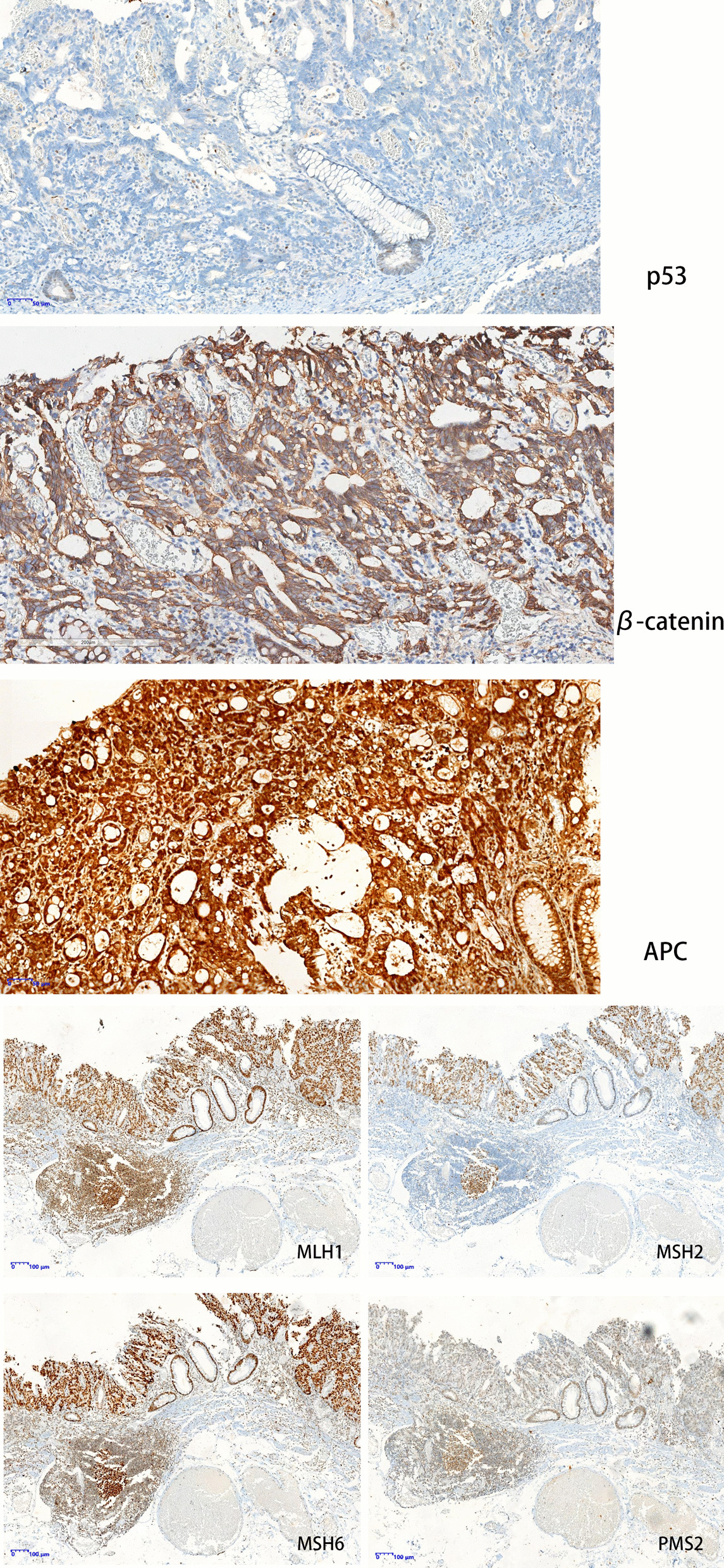
Immunohistochemistry staining of the lesion

A 49-year-old man was referred to our hospital for endoscopic therapy of a lateral spread tumor (LST) in the rectum in December 2019. At the previous hospital, biopsy histology showed high-grade intraepithelial neoplasia (HGIN). He had a three-week history of intermittent hematochezia. Family history for colorectal malignancy was negative. Physical examinations and routine laboratory tests revealed no abnormalities. Before resection, the patient underwent a magnifying chromoendoscopy examination. White-light endoscopy showed a superficially elevated lesion with slight depression in the central part, together with a reddish scar due to previous biopsy (Fig. 1A). The proximal part of the lesion presented with dense irregular microvessels in NBI mode (Fig. 1B). In magnifying endoscopy combined with 0.05% crystal violet staining, the proximal part showed irregular microvessels and turned out to be poorly stained with predominantly non-structural pit pattern, while the background mucosa showed regular Type-I pit patterns according to the Kudo’s classification (Fig. 1C). The distal part showed poorly stained with predominantly non-structural pit pattern, as well as a few small roundish pits scattered over the surface (Fig. 1D). The demarcation line between the lesion and normal mucosa was clearly visible. The whole lesion was lifted after submucosal injection and then resected completely (Fig. 2A) through endoscopic submucosal dissection (ESD). Histology of the resected sample showed poorly differentiated adenocarcinoma invading into deep submucosal layer, with negative lymphovascular invasion and negative resection margin (Fig. 2B–D). P53 Immunohistochemistry staining showed complete absence in the cancerous area, which was predictive of TP53 truncated mutation. The MMR and APC genes showed intact expression, and β-catenin was expressed in the cellular membrane and cytoplasm (Fig. 3). KRAS gene mutation was conducted through Polymerase Chain Reaction (PCR) and also showed negative results. The patient then underwent additional surgery with lymph node dissection and final histology showed no residual tumor and no lymph node involvement. Follow-up surveillance colonoscopy and contrast enhanced computed tomography were performed for the patient in both the first year and second year after surgery. Neither local recurrence nor distant metastasis was detected over a two-year follow-up period.

## Discussion and conclusions

Endoscopic resection is indicated for Tis or T1 tumors and pathological findings of unfavorable features including poorly differentiation and deep submucosal infiltration are considered to be non-curative [1]. Magnifying endoscopy with chemical dye staining is usually conducted for pre-resection assessment in these cases. Kudo’s pit pattern classification, which shows the relationship between pit patterns and histology, is accurate in differentiating neoplastic and non-neoplastic lesions and predicting tumor invasion depth. However, there has been no endoscopic diagnosing criteria in determining histologic type and tumor degree of differentiation for colorectal cancers. Reviewing the literature, we found a few case reports clarifying the endoscopic features of early-stage signet ring cell carcinoma in the colorectum [2–4]. To the best of our knowledge, there have been no reports on the magnifying nor the chromoendoscopic findings of poorly differentiated colorectal adenocarcinoma.

Ohnita et al. [2] reported a primary signet ring cell carcinoma detected at an early stage. As they reported, the margin of the lesion showed IIIL and V_I_ pit patterns, while the central part of the lesion showed V_I_ pit pattern and dense mucus. Similar findings have been reported by Fu et al. [3]. As they explained, signet ring cells preferred to produce mucus so such lesions were difficult to stain and showed avascular areas. However, there was no obvious mucus in our case and the whole lesion was also difficult to stain using either indigo carmine or crystal violet. In our case, the histology revealed a large number of tumor cells overgrowing and loss of normal surface epithelium and crypt-like structure in the mucosal layer. These findings may explain why the lesion was poorly stained. In some histologic sections we observed a few normal glandular ducts surrounded by tumors cells (Fig. 2D), which was consistent with the scattered roundish pits, i.e., Type-I pit patterns in magnifying endoscopy. Minamide et al. [4] reported similar findings in colorectal signet ring cell carcinoma but the lesion was residual after cold snare polypectomy and the diagnosing information may be not adequate. In Kudo’s classification, Type V_N_ pit-pattern refers to loss or decrease of pits with an amorphous structure and indicates invasive submucosal colorectal cancer. Usually, Type V_N_ pit-pattern co-exists with Vi pit-pattern or scratch sign. The lesion in our case presented poorly stained feature with predominately non-structural pit pattern and a few roundish pits scattered on the surface. No obvious Vi pit-pattern or scratch sign was found. These features were different from those of typical Type V_N_ pit-pattern in Kudo’s classification. We confused at the failing to stain the lesion in the beginning and we repeated several times and the outcomes turned out to be the same. Besides the poorly stained feature, the proximal part of the lesion showed irregular microvessels similar to corkscrew vessels which indicated poorly differentiated cancer in the stomach.

This was the first time we encountered with early poorly differentiated colorectal cancer and due to the lack of adequate knowledge, we initially performed endoscopic submucosal dissection for the patient. Non-curative endoscopic treatment resulted in increases in time and cost and decreased patient’s satisfaction. From our case, we suppose that poorly stained features with predominantly non-structural pit pattern in magnifying endoscopy with crystal violet staining may be related to poorly differentiation and thus be inappropriate for endoscopic resection. More researches are needed to make a definite conclusion.

At the same time, we also analyzed the molecular features of the lesion. Colorectal cancers are heterogenous at the genetic level and develop via accumulation of genetic molecular alterations, in which APC, KRAS, TP53 mutations are mostly founded. Several pathways have been proposed for the development and progression of colorectal cancer, including the widely accepted adenoma-carcinoma sequence, the serrated neoplasia pathway, and de-novo carcinogenesis [5]. By definition, de-novo lesions are characterized by the lack of any adenomatous remnant. In our case, the lesion presented with nonpolypoid growth pattern and no adenomatous remnant was founded. Immunohistochemical and molecular analysis of the lesion implied p53 mutation, without any of APC, β-catenin, or KRAS mutation. Thus, we suppose that the cancerous lesion in our case is associated with p53 mutation, in accordance with molecular changes of de-novo colorectal cancers.

## Data Availability

All data generated or analysed during this study are included in this published article.

## Abbreviations

LST: Lateral spread tumor
HGIN: High-grade intraepithelial neoplasia
NBI: Narrow band imaging
ESD: Endoscopic submucosal dissection
P53: Protein 53
TP53: Tumor protein 53
MMR: Mismatch repair proteins
MSH2: MutS homolog 2
MSH6: MutS homolog 6
MLH1: MutL homolog 1
PMS2: Postmeiotic segregation increased 2
APC: Adenomatous polyposis coli
KRAS: Kirsten rat sarcoma viral oncogene homolog
PCR: Polymerase chain reaction
